# Antifungal drug-resistance mechanisms in *Candida* biofilms

**DOI:** 10.1016/j.mib.2022.102237

**Published:** 2022-11-24

**Authors:** Jaspreet Kaur, Clarissa J Nobile

**Affiliations:** 1 Department of Molecular and Cell Biology, School of Natural Sciences, University of California Merced, Merced, CA, USA; 2 Health Sciences Research Institute, University of California Merced, Merced, CA, USA

## Abstract

Infections caused by the *Candida* species of human fungal pathogens are a significant medical problem because they can disseminate to nearly every organ of the body. In addition, there are only a few classes of antifungal drugs available to treat patients with invasive fungal infections. *Candida* infections that are associated with biofilms can withstand much higher concentrations of antifungal drugs compared with infections caused by planktonic cells, thus making biofilm infections particularly challenging to treat. *Candida albicans* is among the most prevalent fungal species of the human microbiota, asymptomatically colonizing several niches of the body, including the gastrointestinal tract, genitourinary tract, mouth, and skin. Immunocompromised health conditions, dysbiosis of the microbiota, or environmental changes, however, can lead to *C. albicans* overgrowth, causing infections that range from superficial mucosal infections to severe hematogenously disseminated infections. Here, we review the current knowledge of antifungal drug-resistance mechanisms occurring in *Candida* biofilms.

## Introduction

Of the estimated 1.5–5.1 million species of fungi on Earth, only about 400 are known to cause disease in humans [17,36]. These pathogenic fungi are responsible for approximately one billion infections and more than 1.6 million deaths annually worldwide [44]. Infections caused by the *Candida* species of human fungal pathogens are responsible for the majority of the reported global deaths by fungi [44]. Over the past few decades, concomitant increases in the immunocompromised population and in the use of antifungal drugs have led to a precipitous increase in the number of infections caused by *Candida* species [7,44].

*Candida albicans* is one of the most prevalent commensal fungal species of the human microbiota, asymptomatically colonizing numerous body sites, including the gastrointestinal tract, genitourinary tract, and skin of healthy individuals [20,37,45]. Changes in host immunity, stress, resident microbiota, and other factors can lead to *C. albicans* overgrowth, causing a wide range of infections ranging from superficial mucosal to severe hematogenously disseminated candidiasis. To date, most studies of *C. albicans* have been carried out in suspension (planktonic) cultures, however, the medical impact of *C. albicans* depends on its ability to form a biofilm, a closely packed community of cells encased in an extracellular matrix [3]. Biofilms are notorious for growing on implanted medical devices, such as catheters, pacemakers, dentures, and prosthetic joints, which provide a surface and sanctuary for biofilm growth [27]. In addition to these abiotic surfaces, biofilms can also grow on biotic surfaces, such as mucosal epithelial linings [28,48].

Unlike the diversity of antibiotic drug classes available for use against bacterial pathogens, there are only three major classes of antifungal drugs used to treat invasive fungal infections: azoles, echinocandins, and polyenes. Azoles (e.g. fluconazole) are the most prescribed class of antifungal drugs used to treat both systemic and superficial fungal infections, polyenes (e.g. amphotericin B) are the oldest class of antifungal drugs used to treat severe systemic fungal infections, and echinocandins (e.g. caspofungin) are the newest class of antifungal drugs typically used to treat recalcitrant fungal infections.

## Drug resistance in *Candida* biofilms

A large body of research in the field has focused on understanding the mechanisms of acquired antifungal drug resistance and tolerance in planktonic (i.e. free-floating) *Candida* cells, and several excellent reviews have been recently published on this topic [10,24]. The major planktonic antifungal drug-resistance and tolerance mechanisms include efflux pump overexpression after exposure to antifungal drugs, mutations in genes that encode drug target enzymes (e.g. *ERG11*), loss-of-function mutations in genes in the ergosterol biosynthesis pathway (e.g. *ERG3*), and mutations in *FKS1*, which encodes 1,3-β-glucan synthase. These acquired planktonic-resistance mechanisms can be shared between both planktonic and biofilm states. However, we know that when the same planktonic strains are grown as biofilms, these resistance mechanisms only account for a proportion of the resistance observed in biofilms. Thus, existing as a biofilm affords *Candida* species with additional resistance and tolerance mechanisms that are unique and specific to the biofilm state. In the following sections of this review, we focus on the mechanisms of antifungal resistance and tolerance that are specific to biofilms.

**Figure 1:**
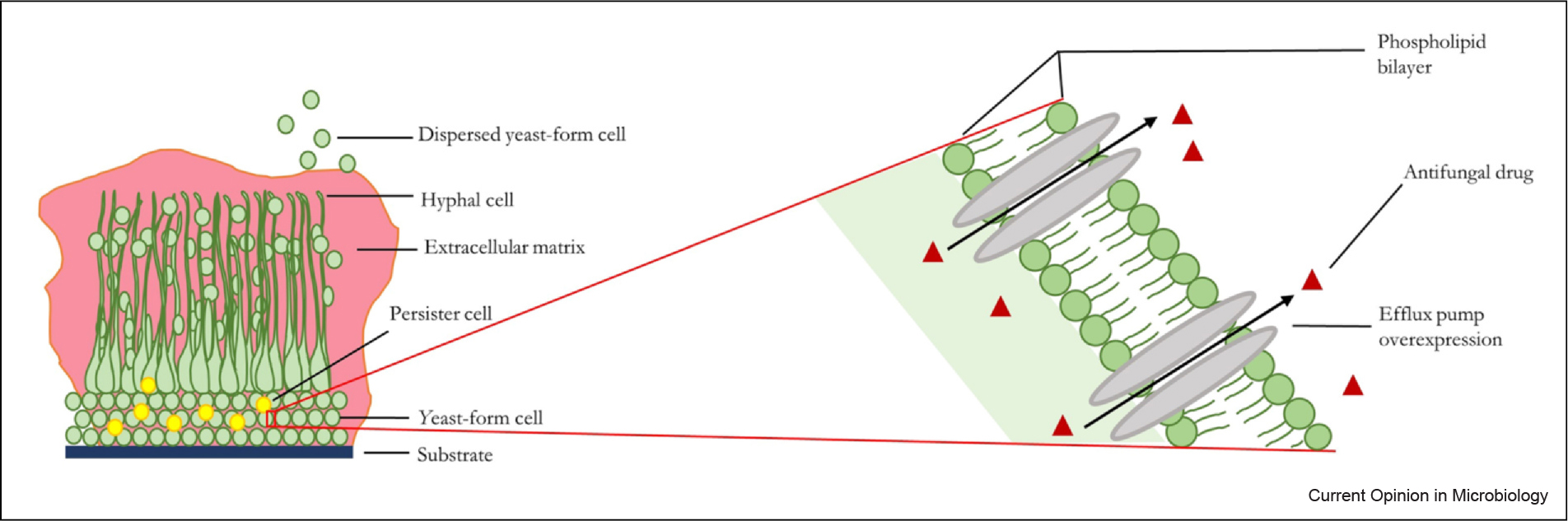
Major drug-resistance and/or tolerance mechanisms in *C. albicans* biofilms. A schematic representation of the major *C. albicans* biofilm drug-resistance and/or tolerance mechanisms: the presence of the extracellular matrix, the upregulation of drug-efflux pumps, and the presence of persister cells.

*C. albicans* biofilms are inherently resistant to the majority of known antifungal drugs, making biofilm infections particularly challenging to treat. Resistance and tolerance of *C. albicans* biofilms to antifungal drugs is multifactorial and mechanistically complex, but is largely attributed to (i) the presence of the extracellular matrix, (ii) the upregulation of drug-efflux pumps (independent of exposure to antifungal drugs), and (iii) the presence of persister cells (Figure 1). We next discuss these three biofilm-specific drug-resistance mechanisms in detail and briefly discuss other mechanisms that are implicated in biofilm-specific drug resistance and tolerance, such as increased cell density and quorum sensing, and the upregulation of the general stress response.

### The extracellular matrix

The *C. albicans* biofilm extracellular matrix is composed of proteins (55%), carbohydrates (25%), lipids (15%), and extracellular DNA (5%), and is a major contributor to antifungal drug resistance and tolerance [32]. The matrix acts both as a physical barrier to drug penetration and supports the overall architecture of the biofilm. Glucans and mannans in the *C. albicans* biofilm matrix, for example, are known to form a complex that sequesters antifungal drugs, significantly increasing the drug tolerance of biofilms compared with planktonic cells [8,30]. Glucan synthesis by Fks1 is a known mechanism that contributes to *C. albicans* biofilm antifungal drug resistance, and it has been shown that increased expression of *FKS1* impacts the susceptibility of *C. albicans* biofilms to antifungal drugs by increasing the levels of biofilm matrix sequestration, thus preventing the antifungal drugs from reaching their cellular targets [33]. Interestingly, this specific Fks1-mediated resistance mechanism appears to be biofilm-specific since modulating *FKS1* levels in *C. albicans* cells grown planktonically, had no impact on antifungal drug susceptibility [33]. We note, however, that several mutations in short conserved regions of *FKS1* have been linked to caspofungin resistance in clinical *C. albicans* isolates grown under planktonic conditions [2]. In addition, glucanases, such as Bgl2 and Xog1, have been shown to be required for assembly of the polysaccharide components of the extracellular matrix and have known roles in antifungal drug sequestration [46]. The importance of matrix glucans and mannans in contributing to biofilm drug tolerance appears to be a conserved mechanism that is also known to occur in biofilms formed by other *Candida* species, such as *Candida glabrata, Candida parapsilosis*, *Candida tropicalis*, and *Candida auris* [8,9]. Notably, biofilms are typically polymicrobial in host settings, forming complex biofilms with many different species, which ultimately changes the composition of the matrix. *C*. *albicans* can form polymicrobial biofilms with several other *Candida* species as well as with bacteria, such as *Staphylococcus aureus*, *Escherichia coli*, and *Streptococcus mutans*, which can further alter the drug sequestration abilities of these complex biofilms [6,12,16,18,49].

Recent work has shown that *Candida* species release extracellular vesicles during biofilm formation that transport components of the extracellular matrix, including glucan and mannan, throughout the biofilm structure [30,50–52]. Interestingly, individual vesicle cargo proteins were shown to play roles in drug sequestration via the glucan–mannan complex of the extracellular matrix. For example, the *tos1*Δ/Δ strain showed enhanced antifungal susceptibility in *C. albicans*, *C. tropicalis*, *C. parapsilosis*, *C. glabrata*, and *C. auris* relative to its complemented strain [50,51]. These findings indicate that extracellular vesicles play important roles in contributing to drug tolerance of *Candida* biofilms, and could be a novel target for future antifungal drug therapies.

Last, the composition of the biofilm matrix is thought to impact how *C. albicans* interacts with the host innate immune system. For example, a *pmr1*Δ/Δ strain, which is unable to produce matrix mannan, forms biofilms that are more susceptible to neutrophil killing than the reference strain [15]. In addition, the release of host neutrophil extracellular traps (NETs) was increased in scanning electron micrographs of biofilms formed by the *pmr1*Δ/Δ strain compared with the reference strain, further supporting the idea that matrix composition contributes to the immune evasion properties of *Candida* biofilms [15].

### Upregulation of drug-efflux pumps

There are two major classes of efflux pumps that modulate drug exportation in *C. albicans*: the ATP-binding cassette superfamily containing Cdr1/Cdr2 transporters, regulated by the Tac1 transcriptional regulator, and the major facilitator class containing Mdr1 transporters, regulated by the Mrr1 transcriptional regulator. These efflux pumps are typically upregulated in planktonic cells only when they are exposed to antifungal drugs, however, in biofilms, these efflux pumps are highly upregulated during the adherence stage of biofilm formation and remain upregulated as the biofilm matures, irrespective of whether or not an antifungal drug is present [11,34,39]. It seems likely that this early upregulation of *C. albicans* drug-efflux pumps may play an important role in the establishment of the biofilm within a host niche and may have evolved as an adaptive response to inhibitory compounds produced by competing microorganisms within the host environment.

### Persister cells

Persister cells are metabolically dormant variants of microbial cells that form stochastically in microbial populations and are highly tolerant to antimicrobial drugs [22,25,26]. In *C. albicans* biofilms, persister cells are thought to form upon surface attachment and exist primarily in the depths of the biofilm basal layer as a small subset of the yeast-form cell population [23,35]. The presence of persister cells within *C. albicans* biofilms was first discovered when biofilms were treated with amphotericin B, and a biphasic fungal cell death response was observed [22,29]. The antifungal drug tolerance observed for persister cells is due to the metabolically dormant state of the cells and is independent of drug-efflux pump expression and cell membrane or cell wall composition. Interestingly, patients suffering from long-term *Candida* infections showed significantly higher persister cell frequency than those with intermittent *Candida* infections, suggesting that persister cells may be important players in the development of recurrent infections [23].

### Other mechanisms contributing to drug resistance and/or tolerance in *Candida* biofilms

In the biofilm environment, cells are densely packed together and communicate with one another via quorum sensing in a cell density-dependent manner. The presence of closely packed cells in a *C. albicans* biofilm leads to increased drug tolerance because the high number of cells in the community protects cells located deeper within the biofilm architecture from exposure to antifungal drugs. Farnesol and tyrosol are two quorum sensing molecules that exert opposing physiological effects on *C. albicans* cells. Farnesol has been shown to inhibit both hyphal formation and biofilm development, while tyrosol has been shown to stimulate adherence and hyphal production in the early stages of biofilm formation [1,5,13,40,43]. Treatment of *C. albicans* cells with farnesol has been reported to induce global gene expression changes, including the activation of drug-resistance genes through the two-component-signaling histidine kinase Chk1 [4,38,43]. Interestingly, treatment of *C. parapsilosis* cells with tyrosol has been reported to lead to the upregulation of the drug-efflux pump genes *CDR1* and *MDR1*. Despite these findings that farnesol and tyrosol upregulate a subset of genes involved in drug resistance, because farnesol and tyrosol have such significant effects on the physiology of fungal cells, they have nevertheless been explored as potential antifungal therapies [14,31,43]. Currently, the utility of farnesol and tyrosol as antifungal therapies is inconclusive and controversial, and additional studies are needed to mechanistically understand the complex physiological effects of farnesol and tyrosol, and of quorum sensing more generally, on *Candida* biofilms.

The upregulation of general stress responses in *Candida* biofilms can result in increased drug resistance in biofilms [41]. Surface contact of *C. albicans* cells results in the activation and accumulation of Mkc1, the terminal mitogen-activated protein kinase of the protein kinase C pathway that is normally activated in response to cell wall stress [19]. *C. albicans* biofilms formed by the *mkc1*Δ*/*Δ strain are abnormally structured and contain fewer hyphal cells compared with the wild-type strain, and the cells of the *mkc1*Δ*/*Δ strain are 100 times more susceptible to fluconazole than the wild-type strain [19,42]. Mkc1 is known to be involved in resistance to azoles and echinocandins, which is mediated by Hsp90 and calcineurin. Indeed, pharmacological inhibition of calcineurin or Hsp90 is synergistic with fluconazole against *C. albicans* biofilms [21,47]. Based on these findings, the Mkc1 contact-activated signaling pathway may be an interesting pathway to explore in the search for novel antibiofilm drug targets.

## Concluding remarks

There are no biofilm-specific drugs in the market that effectively treat biofilm infections in patients. The resistance and tolerance of fungal biofilms to standard antifungal drugs is complex and multifactorial. Biofilms provide physical protection from antifungal drugs (e.g. via the production of the extracellular matrix) and cells in biofilms are intrinsically resistant to antifungal drugs due to their constitutive upregulation of drug-efflux pumps and their altered metabolic states (e.g. via metabolically inactive persister cells). Deeper mechanistic understanding of these and other unique biofilm properties will be important in the future development of antifungal drugs with efficacy against biofilms. In addition, since biofilms are typically polymicrobial in nature, future studies will also need to extensively explore how different microbial species work together to form biofilms that are associated with common infections in patients, and identify novel drug targets for polymicrobial biofilm infections.

## Data Availability

No data were used for the research described in the article.
